# Deferiprone protects against photoreceptor degeneration by inhibiting parthanatos

**DOI:** 10.1038/s41419-025-07686-x

**Published:** 2025-05-19

**Authors:** Beatriz Villarejo-Zori, Juan Zapata-Muñoz, Elena Sierra-Filardi, Ignacio Ramírez-Pardo, Lambert Montava-Garriga, Ian G. Ganley, Patricia Boya

**Affiliations:** 1 Department of Cellular and Molecular Biology, Centro de Investigaciones Biológicas Margarita Salas, CSIC, Madrid, Spain; 2 Department of Medicine and Life Sciences, Universitat Pompeu Fabra (UPF), CIBERNED, Barcelona, 08003 Spain; 3 MRC Protein Phosphorylation and Ubiquitylation Unit, University of Dundee, Dundee, DD1 5EH UK; 4 Department of Neuroscience and Movement Science, University of Fribourg, Chemin. du Musée 14, 1700 Fribourg, Switzerland

**Keywords:** Macroautophagy, Autophagy, Apoptosis

## Abstract

Photoreceptor degeneration is the hallmark of retinitis pigmentosa. Identifying general mechanisms underlying photoreceptor cell death is key to developing effective, mutation-independent treatments to prevent vision loss. Mitophagy is a protective pathway that prevents age-dependent vision loss and is upregulated by iron chelators such as deferiprone (DFP). Therefore, we aimed to investigate the ability of DFP to protect against retinal degeneration via mitophagy. First, we treated mitophagy reporter mice with MNU, a classic inducer of photoreceptor degeneration. MNU induced retinal degeneration and comprehensively inhibited mitophagy, while also inducing lysosomal basification and lysosomal membrane permeabilization. Although DFP rescued cells and retinal explants from the toxic effects of MNU, this effect was independent of mitophagy. Further investigation revealed that PAR polymers accumulation associated with parthanatos cell death was reduced to similar extents by DFP and the PARP inhibitor olaparib. In conclusion, iron chelation can protect against MNU-induced photoreceptor degeneration in retinal explants via parthanatos inhibition.

**Figure:**
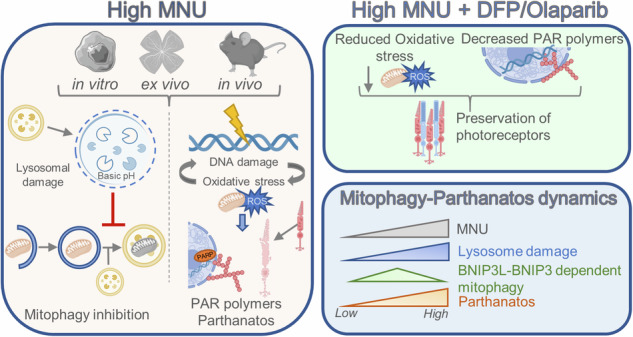
**Olaparib and DFP rescue parthanatos induced cell death after MNU-induced retinal degeneration**. High doses of MNU induce lysosomal damage and mitophagy inhibition. In addition, MNU produces DNA damage and increases oxidative stress, resulting in PAR polymer formation and retinal degeneration (orange panel). DFP and Olaparib are able to rescue retinal degeneration downstream of lysosomal damage (green panel). Sub-lethal doses of MNU induce a peak in mitophagy that is BNIP3L-BNIP3 dependent (blue panel).

**Olaparib and DFP rescue parthanatos induced cell death after MNU-induced retinal degeneration**. High doses of MNU induce lysosomal damage and mitophagy inhibition. In addition, MNU produces DNA damage and increases oxidative stress, resulting in PAR polymer formation and retinal degeneration (orange panel). DFP and Olaparib are able to rescue retinal degeneration downstream of lysosomal damage (green panel). Sub-lethal doses of MNU induce a peak in mitophagy that is BNIP3L-BNIP3 dependent (blue panel).

## Introduction

Retinitis pigmentosa (RP) is one of the main inherited retinal dystrophies worldwide. More than 3000 mutations in around 70 different genes have been detected to produce the disease. Characteristic retinal degeneration manifestations include night vision loss, reduced visual field, and, in some cases, total blindness [1]. Currently, no cure for RP exists. To develop widely applicable treatments for this highly heterogeneous disease it is crucial to identify common pathophysiological features across animal models and to better understand the process underlying photoreceptor degeneration and death.

While photoreceptor cell death in RP has classically been described as apoptosis, recent evidence suggests that parthanatos [2], a non-apoptotic cell death mechanism with high levels of poly(ADP ribose) polymerase-1 (PARP1) activity [3], may in fact drive this process. The PARP inhibitor olaparib rescued cell death in the classic *rd1* mouse model of RP [4]. Moreover, PARP1 is highly activated by DNA strand nicks and plays a role in DNA repair by catalyzing the attachment of PAR polymers to acceptor proteins using NAD+ as a donor of ADP ribose [5, 6]. PARP1 hyperactivation can lead to NAD+ depletion and the accumulation of PAR polymers and poly(ADP-ribosyl)ated (PARylated) proteins in mitochondria, ultimately causing Δψm dissipation and mitochondrial outer membrane permeabilization and triggering mitochondrial release of apoptosis-inducing factor (AIF). Following translocation into the nucleus, AIF mediates chromatin condensation and large-scale DNA fragmentation [3, 7]. Other studies have suggested that ferroptosis, an iron-dependent lipid peroxidation–mediated form of cell death [8], may regulate photoreceptor degeneration, as multiple iron chelators have shown protective effects in another classic RP mouse model, *rd10* [9–11]. However, there is a lack of studies of iron-independent ferroptosis inhibitors such as liproxstatin-1 and ferrostatin-1.

Autophagy is a well-known quality control mechanism present in all eukaryotic cells by which cellular components are engulfed within a double-membrane structure, the autophagosome, and degraded inside lysosomes [12]. Mitophagy is the selective degradation of mitochondria using this autophagy machinery, and its therapeutic potential in retinal diseases has been recently discussed [13]. Depending on how mitochondria are recognized by the autophagosome, mitophagy can be ubiquitin-dependent or -independent [14, 15]. The ubiquitin-dependent pathway is frequently PINK-PRKN-dependent. Upon mitochondrial membrane depolarization, PINK1 (PTEN-induced kinase 1) is stabilized at the outer mitochondrial membrane and PRKN is recruited. This leads to the ubiquitination of outer mitochondrial membrane proteins by PRKN and helps sequestosome-like receptors to recognize mitochondria for autophagy degradation [16]. The ubiquitin-independent mitophagy pathway, by contrast, is mainly regulated by mitophagy receptors, which are resident mitochondrial proteins that interact directly with LC3 and GABARAP in the inner membrane of the nascent autophagosome. The expression of certain mitophagy receptors, such as BNIP3L/NIX and BNIP3, is upregulated in conditions of hypoxia or iron scarcity. Iron chelators such as deferiprone (DFP) and deferoxamine are potent inducers of mitophagy [17, 18].

Very recent data published by our group showed that an increase in mitophagy induced by urolithin A can delay age-dependent neurological decline and preserve visual function [19]. Little is known about the mitophagy quality control mechanisms in RP or the processes by which iron chelators protect against retinal degeneration in RP models [9, 10]. We have previously described how mitophagy in the retina is mainly located in the photoreceptor layer [20], suggesting mitophagy is important for photoreceptors fitness. The aim of the present study was to analyze the mitophagy process during retinal degeneration and determine whether iron chelation is capable of protecting against photoreceptor cell death by inducing mitophagy.

Using the methylating agent N-methyl-N-nitrosourea (MNU) to model the primary loss of PR that occurs in RP [21–23]in *mito-*QC reporter mice [20, 24] and murine and human retinal cells, we demonstrated that MNU treatment compromised mitophagy in the retina and that the iron chelator DFP exerted neuroprotective effects in MNU-treated retinal explants. Surprisingly, DFP rescued MNU-induced cell death not by boosting mitophagy but by inhibiting parthanatos-dependent cell death.

## Results

### In vivo MNU treatment induces retinal degeneration, mitochondrial accumulation, and mitophagy blockade

**Fig. 1:**
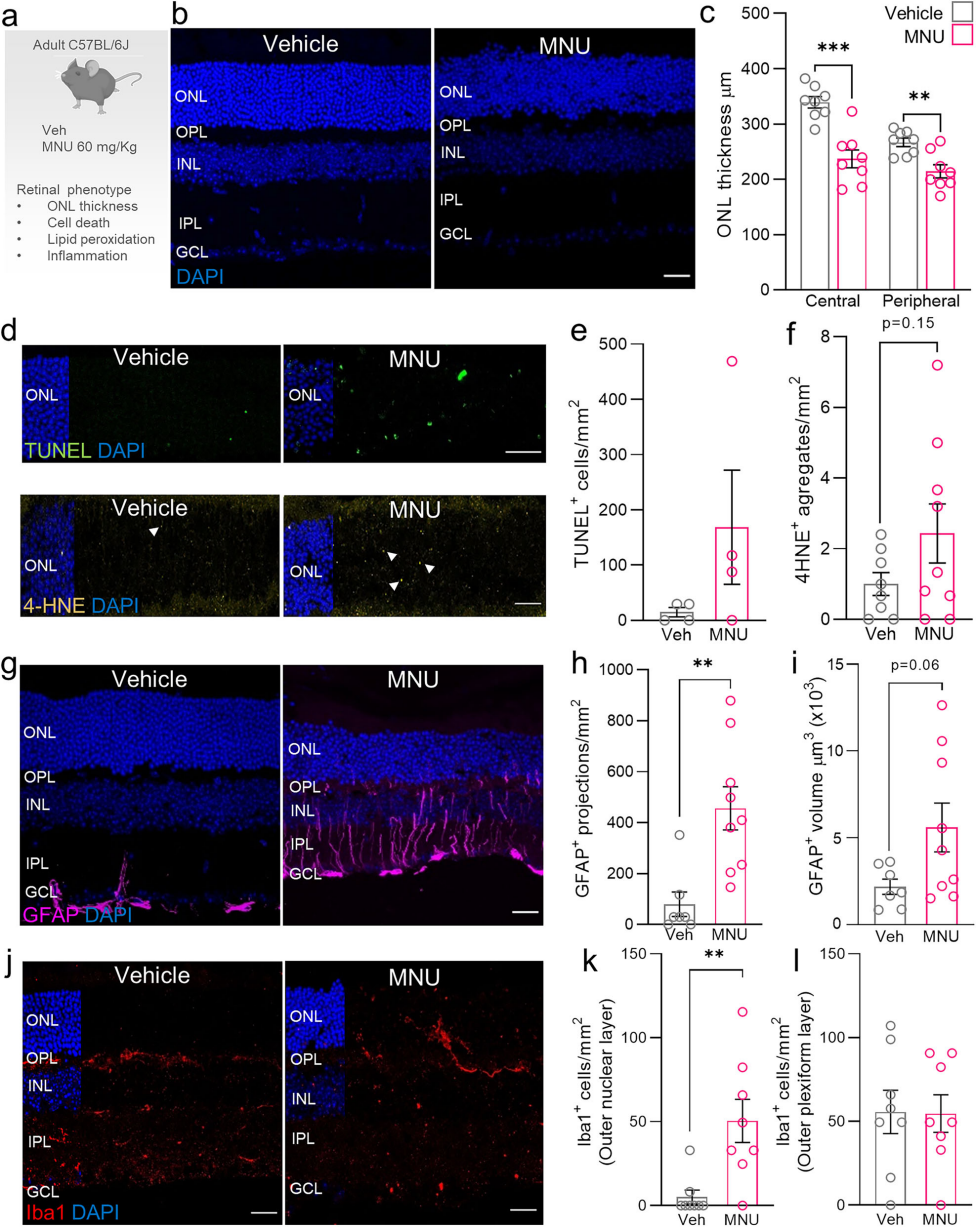
In vivo administration of MNU induces retinal degeneration and inflammation. **a** Experimental design: C57BL/6 J adult mice (*n* = 8–9) were injected intraperitoneally with 60 mg/kg of MNU or vehicle and sacrificed 24 h later. Retinal phenotyping included assessments of ONL thickness, cell death, lipid peroxidation, and inflammation. **b** Representative images from central retina cryosections stained with DAPI (blue). **c** Quantification of ONL thickness in the central and peripheral retina. **d** Representative images and quantification of cell death in the ONL stained with (**e**) TUNEL (green) and (**f**) 4-HNE (yellow) to determine lipid peroxidation. White arrowheads indicate 4-HNE aggregates. **g** GFAP immunostaining (magenta) and quantification of (**h**) glial projections and (**i**) GFAP^+^ volume. **j** Microglia staining with Iba1 (red) and (**k**) quantification of microglia cells in the ONL and (**l**) OPL are shown. Dots represent individual retinas from different mice. All data are expressed as the mean ± SEM. *p < 0.05; **p < 0.01; ***p < 0.001. Two-tailed Student´s *t*-test or two-way ANOVA followed by *post hoc* LSD test for treatment and zone. Scale bars: 25 µm.

Administration of the alkylating agent MNU is classically used to model RP in several species and has been pivotal in unraveling the molecular mechanisms underlying oxidative stress-induced retinal degeneration [25, 26]. We treated C57 mice with 60 mg/kg of MNU and 1 day later analyzed retinal degeneration, oxidative stress, and inflammation-associated parameters (Fig. 1a). MNU-treated animals displayed reduced outer nuclear layer (ONL) thickness and retinal degeneration, which was more prominent in the central retina, indicating early-stage degeneration (Fig. 1b, c). This retinal degeneration was associated with increased TUNEL-positive cells and lipid peroxidation-derived 4HNE-positive aggregates in the ONL (Fig. 1d–f). However, photoreceptor nuclei were positive for TUNEL and negative for activated caspase-3, suggesting activation of a caspase-independent cell death pathway in the retina following in vivo administration of MNU (Fig. 1d, e; Suppl. Fig. 1). MNU-derived cell death resulted in increased glial fibrillary acidic protein (GFAP)-positive astrogliosis compared with vehicle-treated mice (Fig. 1g-i) and increased microglial infiltration as revealed by Iba1 staining (Fig. 1j–l). Together, these data reveal increased levels of oxidative stress, inflammation, and retinal degeneration 1 day after MNU treatment in vivo.

**Fig. 2:**
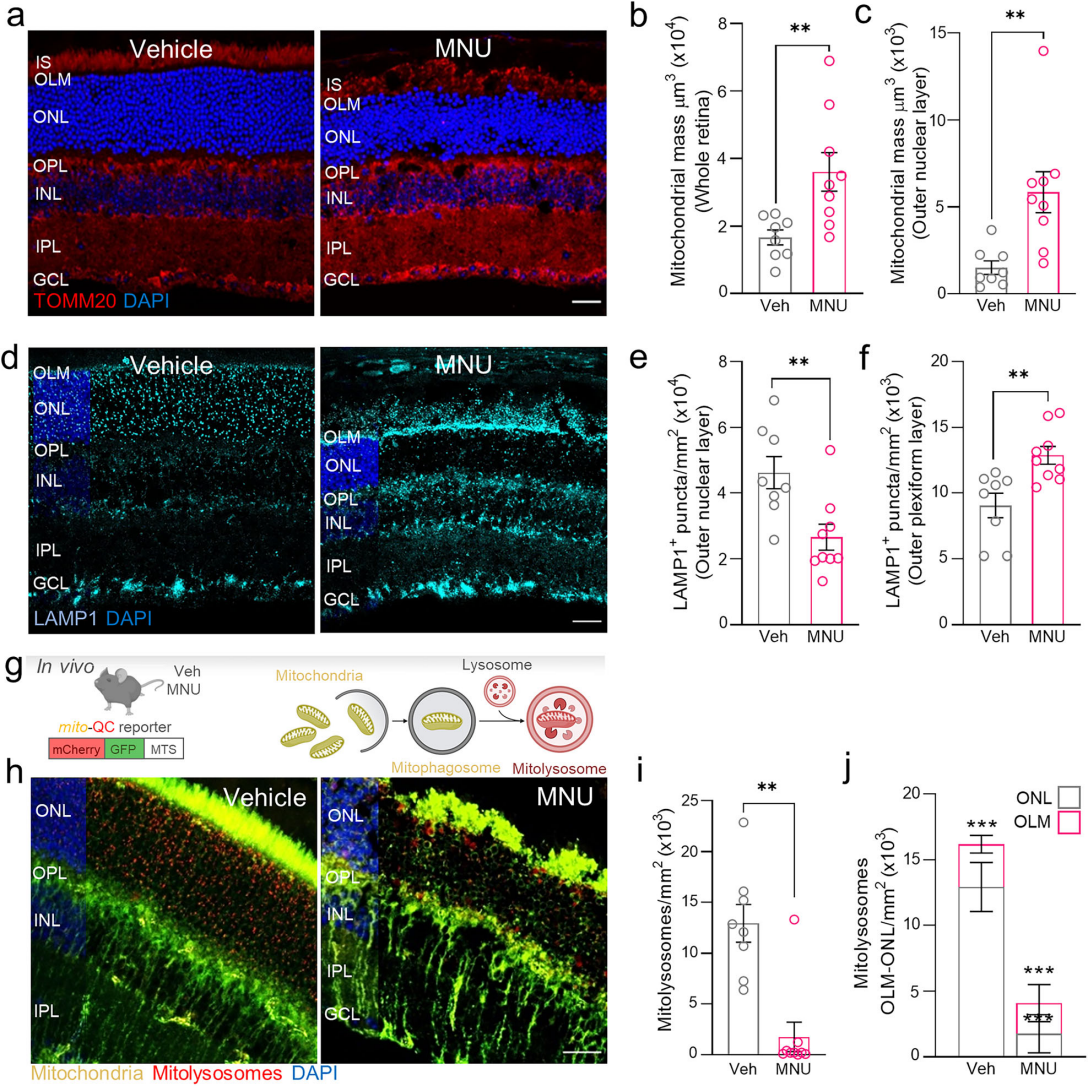
In vivo administration of MNU inhibits mitophagy in photoreceptors. **a** Mitochondria were labeled with anti-TOMM20 (red) in whole-eye retina sections from MNU-treated mice and volume (µm^3^) of mitochondrial mass was quantified in (**b**) the retina and (**c**) the ONL. **d** Representative images of retina cryosections immunostained with the lysosome marker anti-LAMP1 (cyan) and quantification of lysosome number in (**e**) ONL and (**f**) OLM. **g** Adult (3-6 months old) *mito*-QC mice were injected intraperitoneally with MNU (60 mg/kg) or vehicle (*n* = 8–9 per group) and euthanized 24 h later for histological analyses of the retina. Left side of cartoon illustrates the construct carried by *mito-*QC transgenic mice consisting of a mitochondrial outer membrane-localized tandem mCherry-GFP tag, MTS mitochondria targeting sequence. **h** Representative images of *mito-QC* retina cryosections showing mitochondria (yellow) and mitolysosomes (red). Quantification of mitolysosomes in (**i**) ONL and (**j**) OLM. Dots represent individual retinas from different mice. All data are expressed as the mean ± SEM. *p < 0.05; **p < 0.01; ***p < 0.001. Statistical analyses were performed using a two-tailed Student´s *t*-test (for normally distributed data) and non-parametric Mann-Whitney *U* test (for non-normally distributed data). Scale bars: 25 µm.

MNU preferentially alkylates mitochondrial DNA and induces mitochondrial damage [27]. Retinas from MNU-treated mice displayed increased mitochondrial mass compared with those from vehicle-treated mice (Fig. 2a–c). Conversely, lysosomes were massively reduced in the ONL of mice in the MNU group (Fig. 2d, e) and appeared to accumulate near the outer limiting membrane (OLM) (Fig. 2d, f). This increase in mitochondrial mass and parallel lysosomal alterations may be a consequence of impaired mitochondrial turnover via mitophagy. Therefore, changes in mitophagy were investigated using the *mito*-QC reporter mice upon MNU treatment in vivo (Fig. 2g). In these mice, mitolysosomes (mitochondria within lysosomes) can be visualized during mitophagy, as the GFP signal is quenched in the acidic environment of the lysosome (Fig. 2g). Retina degeneration in *mito-*QC mice resulted in a massive reduction in the number of mitolysosomes in the ONL (Fig. 2h, i), which is similarly observed in the lysosomal protease inhibitor leupeptin treatment (Suppl. Fig. 2a-c). Interestingly, we observed accumulation of the few remaining mitolysosomes near the OLM in both MNU- and leupeptin-treated mice (Fig. 2h–j, Suppl. Fig. 2b-d). This same lysosomal phenotype in the retina using MNU (60 mg/kg) and leupeptin suggests lysosomal dysfunction under MNU treatment.

Next, mitolysosomes were visualized in both cones and rods via co-immunostaining with specific antibodies to address which cell type accounted for the reduction in mitolysosomes (Suppl. Fig. 2e-j). According to retinal degeneration, MNU treatment resulted in fewer cones (Suppl. Fig. 2e,g), reduced rod outer segments (Suppl. Fig. 2h,j); and scarce mitolysosomes within both types of photoreceptors. These results point to a reduction in mitophagy flux in photoreceptors as a common process underlying MNU-induced retinal degeneration.

### Mitophagy induced by sublethal MNU doses is BNIP3L/BNIP3 dependent

**Fig. 3:**
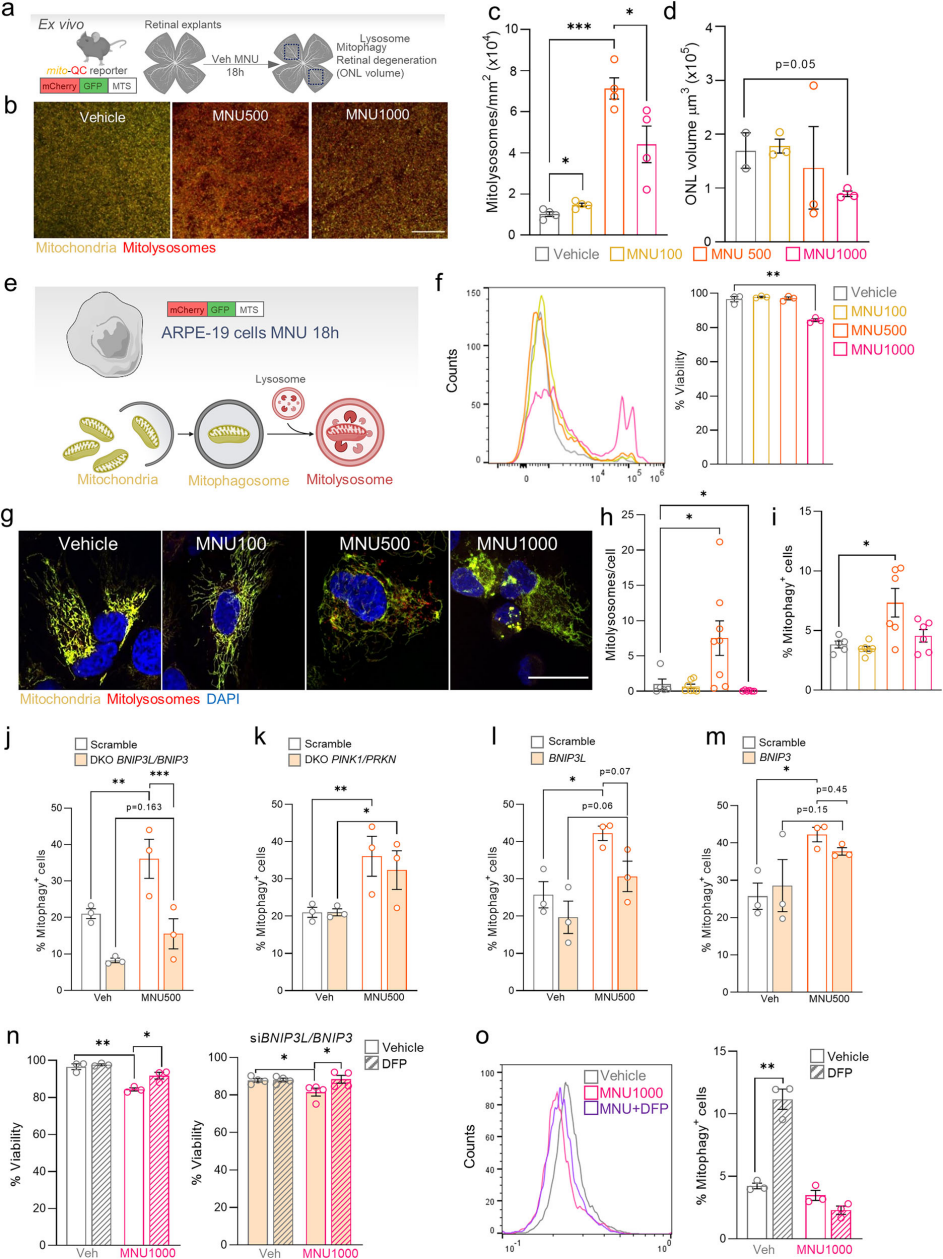
Blockade of mitophagy with high-dose MNU is rescued by DFP in vitro, ex vivo, and in vivo. **a** Experimental design used to examine the effects of MNU in retinal explants (ex vivo): retinas from *mito*-QC mice (*n* = 4 per group) were isolated and cultured with different MNU doses for 18 h. Mitophagy and retinal thickness were quantified in fixed retinal explants. **b** Representative images of *mito-*QC retinal explants treated with different doses of MNU: 100, 500 and 1000 μg/ml and quantification of mitophagy density levels (red dots/mm^2^ retina) (**c**) and volume of ONL (µm^3^) (**d**). **e** Experimental design used to examine the effects of MNU in retinal ARPE-19 *mito*-QC. **f** % cells with and without DAPI in MNU-treated ARPE-19 cells measured by flow cytometry and viability quantification. **g** Representative images of ARPE-19 *mito-*QC cells treated with increasing doses of MNU (left-right) and quantification of mitophagy by (**h**) fluorescence microscopy and (**i**) flow cytometry. **j**–**m** Quantification of mitophagy by flow cytometry in vehicle- and MNU (500 µg/ml)-treated ARPE-19 *mito-QC* cells subjected to downregulation of *BNIP3L/BNIP3, PINK1/PRKN, BNIP3L* and *BNIP3* genes (left to right). **n** Quantification of viability based on DAPI intensity by flow cytometry in ARPE-19 *mito*-QC cells with different treatments (with/without 1000 µg/ml MNU and/or with/without DFP) in basal (left) or BNIP3L/BNIP3 silencing (right) conditions. **o** Quantification of mitophagy by flow cytometry in ARPE-19 *mito-*QC cells in different treatment conditions (with/without 1000 µg/ml MNU and/or with/without DFP) (right), and representative histogram depicting cell populations (left). Dots represent individual mice or different cell experiments. All data are expressed as the mean ± SEM. *p < 0.05; **p < 0.01; ***p < 0.001. Two-tailed Student´s *t*-test (for normally distributed data) and non-parametric Mann-Whitney *U* test (for non-normally distributed data) were used for statistical analyses. Scale bars: 25 µm.

To address the kinetics of mitophagy along photoreceptor cell death we used retinal explants treated with different MNU doses (Fig. 3a, b). As expected, the greatest retinal thickness reduction was observed with the highest MNU dose (Fig. 3d). Interestingly, we observed an increase in mitolysosomes in the ONL of retinal explants with low doses (100 and 500 µg/ml) of MNU; whereas at higher doses (1000 µg/ml), the increase in mitolysosomes observed at the 500 µg/ml dose was reduced (Fig. 3c). This suggests that mitophagy modulation along MNU-induced photoreceptor degeneration could be a key mechanism to preserve retinal integrity.

To decipher the signaling pathways underlying the modulation of mitophagy observed after MNU treatment, we used the retinal pigmented epithelium human cell line ARPE-19, expressing the *mito*-QC reporter (Fig. 3e). First, cell viability was checked with different MNU doses (100-1000 µg/ml), showing lethal effects only at the highest dose (Fig. 3f). Mitolysosomes were then determined by fluorescence microscopy and flow cytometry (Fig. 3g–i) at the different conditions. Sublethal MNU doses induced mitophagy whereas the highest MNU dose resulted in a reduction of mitolysosomes, in accordance with our ex vivo and in vivo data.

We next investigated which pathways mediated the observed induction of mitophagy. Both the PINK1/PRKN and receptor-mediated BNIP3L/BNIP3 mitophagy pathways can be activated in the retina under different conditions [19, 28]. The MNU-dependent increase in mitophagy was abolished when the cells were transfected with siRNA against *BNIP3L* and *BNIP3* (DKO BNIP3L/BNIP3) (Fig. 3j). However, this did not occur when *PINK1* and *PRKN* were downregulated (DKO PINK1/PRKN) (Fig. 3k). Basal mitophagy was decreased in DKO *BNIP3L/BNIP3* cells (Fig. 3j) but remained unchanged following downregulation of *PINK1/PRKN* and downregulation of either BNIP3 or BNIP3L (Fig. 3l,m). These findings suggest some degree of compensation between the different mitophagy pathways [29]. In conclusion, these data show that both basal and MNU-induced mitophagy are *BNIP3L/BNIP3* dependent and when *BNIP3L* is downregulated, mitophagy is regulated to some degree via compensatory BNIP3-mediated signaling.

Based on our findings described above, BNIP3L and BNIP3 regulate both basal and MNU-induced mitophagy, we hypothesized that upregulation of this pathway could potentially rescue MNU-induced cell death. To test this hypothesis, we used DFP, a classical inducer of receptor-mediated mitophagy [18]. DFP is an iron chelator that stabilizes HIF-1 and increases *BNIP3L* and *BNIP3* mRNA expression [17]. While DFP rescued MNU-induced cell death in ARPE-19 cells, the effect was independent of BNIP3L, as DFP-mediated rescue was also observed in cells incubated with siRNA against *BNIP3L/BNIP3* (Fig. 3n). Mitophagy was not increased by DFP in MNU-treated cells, suggesting that DFP rescue is independent of mitophagy after MNU administration (Fig. 3o). In conclusion, our findings show that high doses of the alkylating agent MNU induce cell death, which in turn is rescued by DFP via a mitophagy-independent pathway.

### High MNU doses induce lysosomal basification and lysosomal membrane permeabilization

**Fig. 4:**
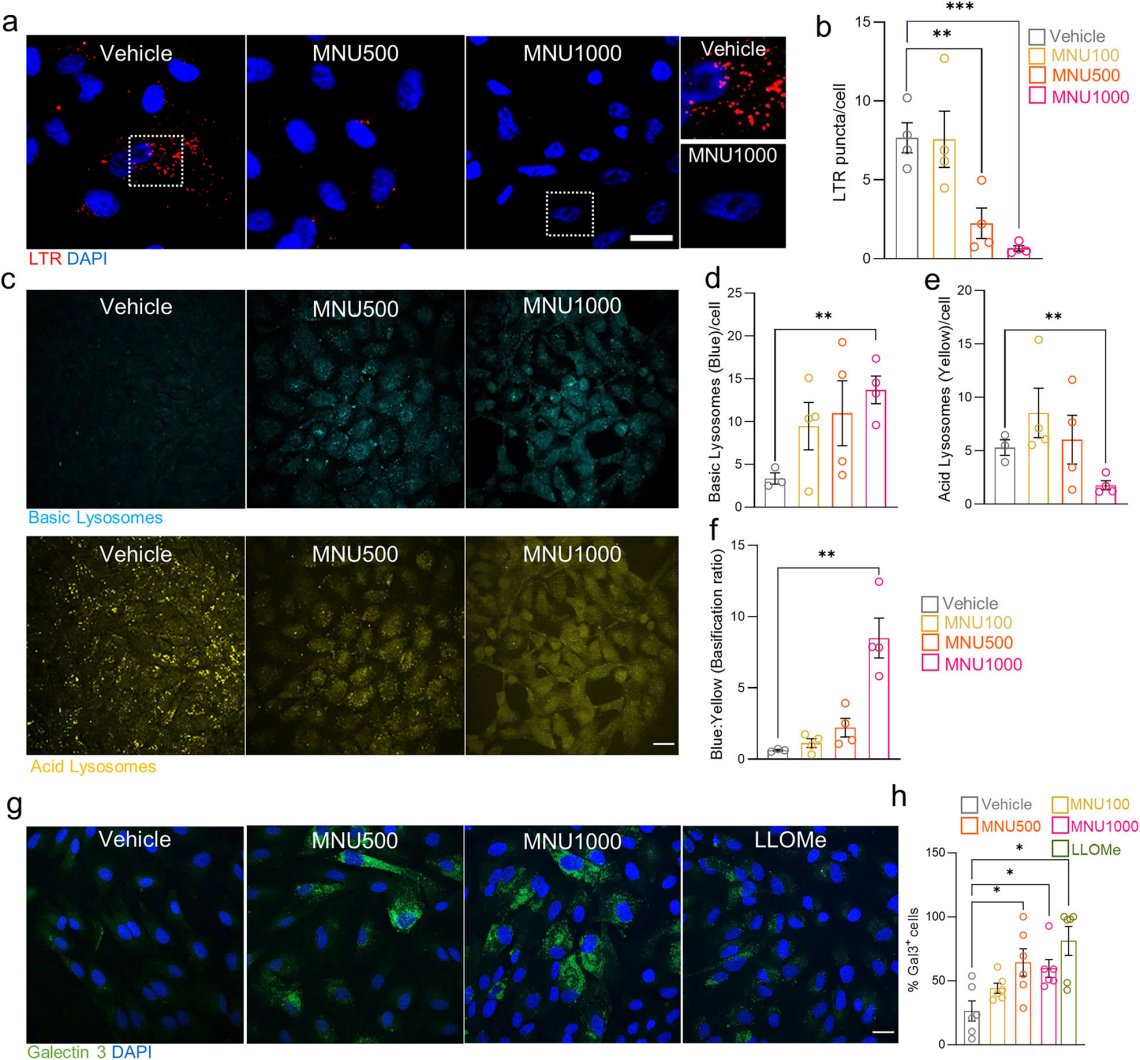
MNU induces lysosomal alterations that are not reversed by DFP. **a** Representative images of ARPE-19 cells treated with different doses of MNU (500 and 1000 μg/ml) and labeled with LysoTracker Red (red, to label acidic lysosomes) and DAPI (blue). Insets show a cell treated with vehicle or 1000 μg/ml MNU (right). **b** Quantification by fluorescence microscopy of acidic lysosomes in cells treated with 100, 500, or 1000 μg/ml MNU. **c** Representative images of ARPE-19 cells treated with different doses of MNU (500 µg/ml, 1000 µg/ml) and labeled with LysoSensor Yellow/Blue DND-160, which allows distinction of basic lysosomes (blue; top) from acidic lysosomes (yellow; bottom). Fluorescence microscopy quantification of basic lysosomes (**d**), acid lysosomes (**e**), and basification ratio (**f**) in cells treated with 100, 500 or 1000 µg/ml MNU. **g** Representative images of ARPE-19 cells treated with 500 or 1000 µg/ml MNU or LLOMe (positive control) and transfected with a fluorescent *Galectin-3* (green) reporter. Cells were also stained with DAPI (blue). **h** % of cells expressing the reporter (Gal3 (+)), detected by fluorescence microscopy, relative to the total number of cells. Dots represent different cell experiments. All data are expressed as the mean ± SEM. *p < 0.05, **p < 0.01; ***p < 0.001; ***p < 0.001. Statistical analyses were performed using a two-tailed Student´s *t*-test. Scale bars: 25 µm.

We investigated whether the inability of DFP to induce mitophagy in the presence of high doses of MNU might be due to alterations in lysosomal function. Since MNU-treated ARPE-19 cells showed reduced LysoTracker Red staining (Fig. 4a, b), we used the LysoSensor Blue/Yellow dye to more precisely measure lysosomal pH. Lysosomes became more alkaline with increasing MNU doses, as evidenced by the increase in blue and decrease in yellow fluorescence (Fig. 4c–e). The ratio between alkaline (blue) and acidic (yellow) conditions clearly demonstrates lysosomal basification after MNU treatment (Fig. 4f).

We next explored whether lysosomal damage might be due to MNU-induced lysosomal membrane permeabilization (LMP), which is often seen in conditions of increased oxidative stress [30]. To assess LMP, we transfected ARPE-19 cells with a fluorescent reporter for LGALS3/galectin-3 to visualize intralysosomal recruitment of cytosolic LGALS3/galectin-3 after LMP [31, 32]. The lysosomotropic agent L-leucyl-L-leucine methyl ester (LLOMe) was used as a positive control. Increasing doses of MNU had a dose-dependent effect on LMP and enabled the intralysosomal recruitment of cytosolic LGALS3/galectin-3 (Fig. 4g, h). Notably, DFP did not reduce galectin-3 staining (data not shown), indicating that it exerts its protective effect either independently or downstream of lysosomal damage. Loss of lysosomal pH stability and integrity upon MNU treatment, especially at higher doses, may partially explain the greater reduction in mitophagy observed during MNU-induced retinal degeneration.

### Iron chelation rescues MNU-induced cell death in retinas and ARPE-19 cells through parthanatos inhibition

**Fig. 5:**
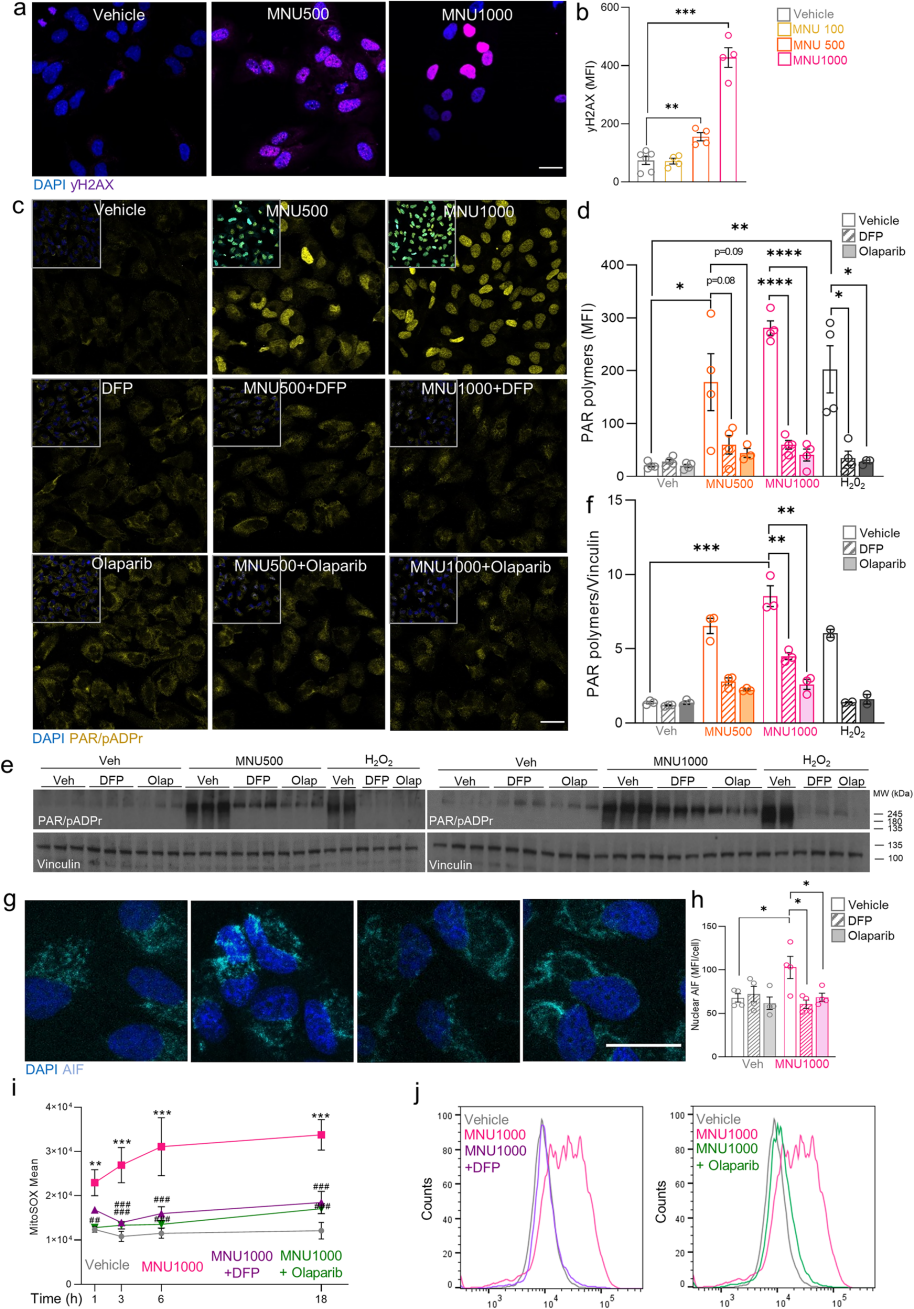
DFP decreases accumulation of PAR polymers, AIF nucleus translocation and oxidative stress associated with MNU-induced parthanatos in ARPE-19 cells. **a** Representative images of ARPE-19 cells treated with different doses of MNU (100, 500, and 1000 µg/ml) for 18 h and labeled with ƴH2AX (purple) and **b** quantification of ƴH2AX labeling by mean fluorescence intensity (MFI). **c** Representative images of ARPE-19 cells treated with different doses of MNU (500, 1000 µg/ml) combined with DFP or olaparib for 10 min and immunolabeled with anti-PAR/pADPr (cyan) to stain PAR polymers, and (**d**) quantification of PAR polymers by MFI in ARPE-19 cells treated with DFP, olaparib, 500 or 1000 µg/ml MNU alone (unfilled bars) or combined with DFP or olaparib (striped bars), using H_2_O_2_ as a positive control. **e** Western blot to detect expression of PAR/pADPr and vinculin (loading control) in protein extracts from ARPE-19 cells treated with DFP or olaparib alone (plain bars) or in combination with 500 (left) or 1000 (right) µg/ml MNU (striped bars) for 10 min. Co-treatment with H_2_O_2_ was used as a positive control. **f** Quantification of PAR polymer protein levels relative to vinculin. **g** Representative images of ARPE-19 cells treated with 1000 µg/ml MNU combined with DFP for 6 hours and immunolabeled with anti-AIF (cyan) to stain AIF nuclear translocation, and (**h**) quantification of nuclear AIF by MFI. **i** ARPE-19 cells were treated with 1000 µg/ml MNU alone (pink line) or in combination with DFP (purple line) or olaparib (green line) and mitochondrial superoxide levels were quantified at different time points (1, 3, 6, and 18 h) by flow cytometry, measuring the MFI parameter of cells labeled with MitoSOX Red Mitochondrial Superoxide Indicator, using H_2_O_2_ as a positive control. **j** Fluorescence intensity of MitoSOX in different cell populations: left, cells treated with vehicle (gray), 1000 µg/ml MNU (pink) or 1000 µM MNU + DFP (purple); right, cells treated with vehicle (gray), 1000 µg/ml MNU (pink) or 1000 µg/ml MNU + olaparib (green). Dots represent different cell experiments. Data are expressed as the mean ± SEM. */^#^*p* < 0.05, **/^##^*p* < 0.01; ***/^###^*p* < 0.001. *: relates versus control. #: relates versus MNU1000. Statistical analyses were performed using a two-tailed Student´s *t*-test (for normally distributed data) and non-parametric Mann-Whitney *U* test (for non-normally distributed data) or two-way ANOVA followed by *post hoc* LSD test to assess the effects of treatment and time. Scale bars: 25 µm.

We next aimed to investigate the mechanism of MNU-induced cell death. Considering that alkylating agents such as MNU induce DNA damage [22, 33], we observed increased γH2AX nuclear staining in MNU-treated cells (Fig. 5a, b). However, observed DNA damage did not result in apoptosis mediated through caspase activation. Alternatively, DNA damage often leads to overactivation of PARP1, which can lead to overproduction of PAR polymers and cell death through parthanatos [34, 35]. This type of cell death can be inhibited by blocking PARP1 activity and triggered by several mutagenic agents similar to MNU [36]. Accordingly, we hypothesized that DFP might rescue MNU-induced cell death by inhibiting parthanatos.

First, we assessed overproduction of PAR polymers upon MNU treatment in ARPE-19 cells. Both doses, MNU500 and MNU1000, triggered the production of these polymers, as evidenced by fluorescence microcopy (Fig. 5c, d) and western blot (Fig. 5e, f). Notably, fluorescence microscopy showed that PAR polymers produced upon MNU treatment were located within the cell nucleus (Fig. 5c). Both DFP and olaparib (a PARP inhibitor) treatments remarkably avoided the PAR polymer overproduction upon MNU treatment (Fig. 5c–f). Indeed, we found that DFP (Fig. 3n) exerted a similar protective effect to the PARP inhibitor olaparib in this setting [37] (Suppl. Fig. 3a). Following the increase in PAR polymers, there is evidence that AIF is then translocated to the nucleus producing large-scale DNA fragmentation. We found indeed increased nuclear translocation of AIF after high MNU dose treatment in ARPE-19 cells and DFP or olaparib were able to reduce this translocation to control levels (Fig. 5 g, h). Moreover, MNU exposure resulted in increased oxidative stress, one of the main triggers of parthanatos [36] which was also restored with both DFP and olaparib interventions (Fig. 5i, j).

**Fig. 6:**
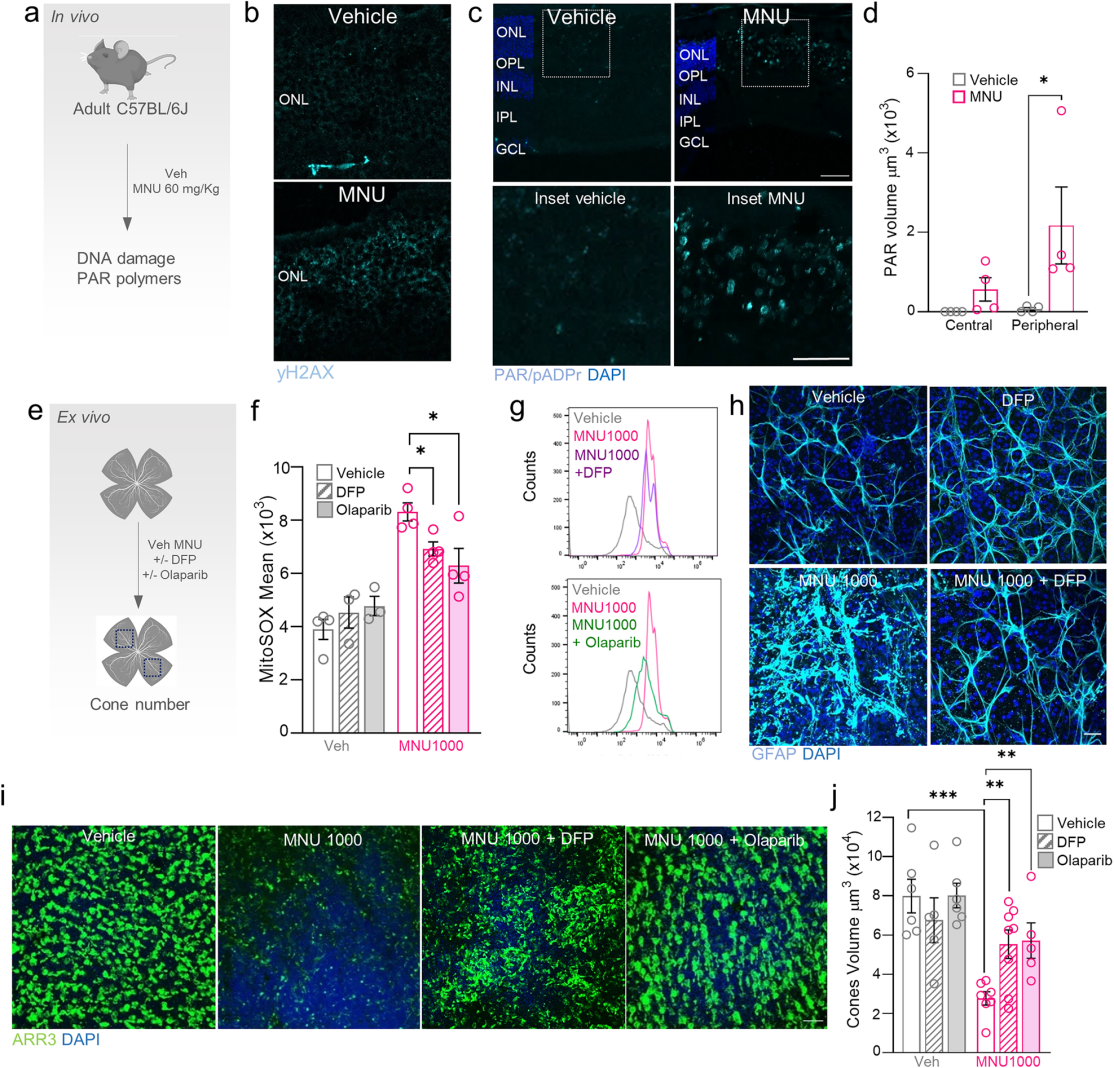
Deferiprone oxidative stress and inflammation and preserves cone viability in ex vivo retinas. **a** Adult (3-6 months old) C57BL/6 J mice were injected intraperitoneally with MNU (60 mg/kg) or vehicle (*n* = 4 per group) and euthanized 24 h later for histological analyses of the retina. Immunostainings to detect DNA damage and PAR polymers in the retina were performed. **b** Representative images of retinal cryosections from mice treated with vehicle or MNU and immunostained with anti-yH2AX (cyan) to label DNA damage. **c** Representative images of retinal cryosections from mice treated with vehicle or MNU and immunostained with anti-PAR/pADPr (cyan) to label PAR polymers and (**d**) quantification of the volume of PAR polymer-positive immunostaining in retinas (central and peripheral areas) in mice treated with vehicle or MNU. **e** Adult (3-6 months old; *n* = 4-8 per group) C57BL/6 J mice were euthanized and retinas were isolated and cultured with the different treatments for 6 h and 18 h. Explants were fixed and processed to study mitochondrial superoxide levels by flow cytometry and retinal thickness and cone number. **f** Retinas were treated with 1000 µg/ml MNU alone or in combination with DFP or olaparib and mitochondrial superoxides were quantified at 6 h by flow cytometry, measuring the MFI parameter of cells labeled with MitoSOX Red Mitochondrial Superoxide Indicator. **g** Fluorescence intensity of MitoSOX in different cell populations: left, cells treated with vehicle (gray), 1000 µg/ml MNU (pink) or 1000 µM MNU + DFP (purple); right, cells treated with vehicle (gray), 1000 µg/ml MNU (pink) or 1000 µg/ml MNU + olaparib (green). **h** Retinas ex vivo were treated with 1000 µg/ml MNU alone or in combination with DFP and immunolabeled with GFAP (cyan). **i** Immunostaining of cone arrestin (ARR3, green) in ex vivo retinas treated with high doses of MNU (1000 µg/ml) with/without DFP or olaparib (striped bars) and (**j**) quantification of the area of cone arrestin-positive labeling. Dots represent individual retinas from different mice. All data are expressed as the mean ± SEM. *p < 0.05, **p < 0.01; ***p < 0.001. Statistical analyses were performed using a two-tailed Student´s *t*-test. Scale bars: 25 µm.

Previous studies have shown that parthanatos is involved in light damage-induced retinal degeneration and several mouse models of RP, such as *rd1* mice [38, 39]. This prompted us to address whether parthanatos might be responsible for MNU-induced retinal degeneration in vivo (Fig. 6a). We found an increase in DNA damage labeled with γH2AX (Fig. 6b) and PAR polymer production (Fig. 6c, d), which was more prominent in the peripheral area of the retina. These results suggest that parthanatos may be a key cell death mechanism accounting for retinal degeneration after MNU treatment.

Finally, we investigated whether the inhibition of parthanatos with olaparib or DFP would rescue MNU-induced oxidative stress and cell death in retinal explants (Fig. 6e). We observed that, as in cells, DFP and olaparib decrease the oxidative stress induced by MNU in the retinas (Fig. 6f, g), and also, DFP decreases inflammation determined with GFAP staining (Fig. 6h). Surprisingly, cone loss associated with MNU-induced retinal degeneration was prevented by either DFP or olaparib treatments (Fig. 6i, j). In conclusion DFP reduced PAR polymers production, mitochondrial oxidative stress and inflammation, leadsing to cone survival.

Altogether, these data provide evidence of the contribution of parthanatos in MNU-induced retinal degeneration and how its inhibition partially rescued photoreceptor cell death. Therefore, we propose a mitophagy-independent protective role of iron chelation agents that may preserve retinal integrity by safeguarding mitochondrial fitness.

## Discussion

Iron chelation has shown neuroprotective effects in retinal dystrophies, including genetic models of RP [9–11]. We have investigated the potential protective effect of the iron chelator DFP in a pharmacological model of PR degeneration by stimulating mitophagy, and observed that DFP protected against MNU-induced cell death both ex vivo and in vitro. Surprisingly, this rescue was independent of selective autophagy, as siRNA for BNIP3L and BNIP3 mitophagy regulators did not abolish DFP-induced survival. In addition, we found that, although low doses of MNU induce BNIP3L/BNIP3-dependent mitophagy, lethal doses of MNU resulted in lysosomal basification and LMP, preventing mitophagy and supporting previous findings by our group in *rd10* mice [40], where it is shown how LMP also occurs in this genetic model.

Parthanatos is the cell death resulting from the activation of PARP1 and the accumulation of PAR polymers. It has been described as a unique pathway, distinct from apoptosis, necroptosis, or any other known forms of cell death [3, 41]. The term “parthanatos” is derived from “PAR”—poly (ADPribose)—and “Thanatos”—the personification of death in Greek mythology. We observed an elevated number of PAR polymers in vivo and in vitro after MNU treatment and found that the general parthanatos inhibitor olaparib rescued photoreceptor cell death. This observation, together with data from other RP animal models, lends support to the involvement of parthanatos in photoreceptor neurodegeneration, irrespective of the source of damage (mutation or genotoxic stress) [2, 42, 43].

Our data show that the production of PAR polymers, and the subsequent AIF translocation, was reduced to similar extents by DFP and olaparib. In our review of the literature, we found just one report of iron chelation-mediated inhibition of PAR synthesis after H_2_O_2_ exposure [44]. Those authors also described a reduction in mitochondrial reactive oxygen species (ROS), DNA damage, and inflammation after iron depletion. Our findings support the link between iron chelation and parthanatos in MNU-treated retinas, as DFP also exerted a protective effect before PARP activation and mitochondrial ROS bursts. We hypothesize that MNU alkylates nuclear and mitochondrial DNA and triggers PARP1 activation in both sites, as this enzyme has been detected also in mitochondria [45]. The energy required for poly(ADP)ribosylation would be provided by NAD+ and ATP [5], potentially generating mitochondrial and nuclear stress through ROS production and creating a cycle of DNA damage. Because iron is known to increase cellular ROS through Fenton reactions [46], we speculate that DFP might rescue cell death by diminishing mitochondrial ROS generation, DNA damage, and PARP activation. Another aspect requiring further exploration is the potential reduction in NAD+ availability, essential for PARP activation, caused by inhibition of mitochondrial complex I and III activity through iron chelation [47]. In fact, a recent study about the metabolic changes after DFP treatment in ARPE-19 cells shows how the mitochondrial electron transport change pathway is decreased in comparison with control cells [18, 48]. Finally, DFP might reduce cell death by chelating intralysosomal iron, which, as reported elsewhere, can reduce lysosomal damage and LMP [49]. This did not appear to occur in our experimental setting, as neither DFP nor olaparib reduced galectin-3 staining after MNU treatment.

The protective effect of DFP on MNU-mediated cell death is supported by other reports in the literature. Green tea extract, for example, which reduces the expression of heme oxygenase 1, protects against MNU-induced cell death, highlighting the important role played by iron in this process (heme oxygenase 1 releases iron from the heme group) [50]. The ROS scavenger edaravone also protects 661 W photoreceptor-derived cells from MNU by reducing lipid peroxidation (4-HNE) and DNA damage measured with 8-hydroxy-2’-deoxyguanosine [51]. Another study found that heat shock protein 70 (HSP70) induction by valproic acid delayed MNU-induced photoreceptor cell death and that this protective effect was abolished by HSP inhibitors. Interestingly, valproic acid is a histone deacetylase (HDAC) inhibitor, and HDAC inhibition protects rods and cones in genetic RP models [52, 53]. The connection between HDAC, PARP, and cell death has been explored extensively elsewhere [54], and it is clearly exposed how the cGMP accumulation characteristic of many genetic RP models can activate HDAC and PARP through protein kinase G (PKG). The involvement of HDAC and PARP activities in both MNU-induced cell death and genetic models of RP strengthens the connections between the processes involved in photoreceptor cell death and highlights the relevance of our study. Another finding linking MNU and genetic models is the detection of hypermethylated DNA at the peak of *rd1* retina degeneration [4]. Rescue with olaparib did not induce any changes, indicating that DNA methylation occurs upstream of PARP1 in both models [4].

Our study highlights the potential ambiguity and challenges arising from the different terms used to categorize types of cell death. We have described an iron-dependent form of cell death involving mitochondrial ROS and lipid peroxidation, features typical of *ferroptosis*. The process, however, was also PARP-mediated and shows AIF translocation and could therefore also be classified as *parthanatos*. Finally, it should be noted that although we propose a novel connection between PARP-mediated cell death and iron, the exact mechanism by which iron reduces PARP activation and mitochondrial ROS in parthanatos requires further investigation, as DFP-mediated PARP inhibition could also be an upstream effect in the cGMP-dependent cell death pathway, as described in previous studies [54].

Given the multiple and highly heterogeneous causes of RP, it is crucial to understand the nature and characteristics of photoreceptor cell death to develop effective, mutation-independent, treatment strategies. The identification of common pathways across animal models for RP could provide valuable insights into the nature of photoreceptor demise. In order to develop treatments spanning the full spectrum of RP, it is first essential to understand how and why photoreceptors die. In this study, we have investigated photoreceptor cell death induced by the alkylating agent MNU and demonstrated a protective effect of iron chelation via inhibition of parthanatos. The development of new PARP inhibitors is an active area of research [55]. Although these compounds are being investigated as potential cancer treatments in clinical trial settings, their ability to maintain cellular bioenergetics and suppress oxidative stress in non-oncological diseases is also being explored [35, 55, 56], and they may also open novel therapeutic avenues in the field of retinal diseases.

## Material and methods

### Animal procedures

All animal experiments were performed following European Union guidelines and the ARVO Statement for the Use of Animals in Ophthalmic and Vision Research. Animal procedures were approved by the CSIC ethical review committee and the Comunidad de Madrid (PROEX 154.3/21). *Mito*-QC mice (constitutive knock-in of mCherry-GFP-mtFIS1^101–153^) were generated in the laboratory of Ian Ganley and bred in the CIB animal facility [24]. Mice used in our studies were homozygous and between 3 and 6 months old. Sample size was not preestimated and for the control and experimental groups there was no specific assignment of mice to one group and the other. Mice were housed at the CIB animal facility in a temperature-controlled barrier facility on a 12-h light/dark cycle, with free access to water and food. Males and females were used indistinctly, and retinas were consistently isolated at the same time of day, after lights were turned on, to avoid confounding effects and variations in autophagy due to circadian rhythms.

For the MNU-induced RP model, 13 mg/ml of MNU solution (TR-M325815; Toronto Chemicals) was freshly prepared sterile in physiological saline [NaCl] 0.9% (999791.5; ERN)-acetic acid 0.05% (1.00063.1000; Merck). Mice received a single intraperitoneal injection of 60 mg/kg MNU (experimental group; *N* = 9) or vehicle (0.9% NaCl-0.05% acetic acid; control group; *N* = 8) and were sacrificed 1 day later. Leupeptin (L2884, Sigma) was prepared fresh at 5 mg/ml before injection by dilution in filtered and sterilized 0.9% NaCl. For in vivo assessment of autophagy flux, mice were injected with leupeptin (40 mg/kg; *N* = 4) or vehicle (0.9% NaCl; *N* = 4) and their retinas were dissected for analysis 18 h later.

### Tissue preparation for imaging

For retina flat mounts, eyes were enucleated and briefly washed in PBS. The optic nerve, cornea, lens and RPE were carefully removed and the resulting posterior eyecup was fixed for 1 h in freshly prepared ice-cold 3.7% (w:v) PFA (171010, EMS) in 200 mM HEPES buffer (4-(2-hydroxyethyl)-1-piperazineethanesulfonic acid; Gibco, 15630-080) at pH 7.0. Four perpendicular incisions were then made on the fixed retina. Tissues were stored in 0.01% azide in PBS at 4 °C until further processing. For cryosections, mouse eyeballs were marked for orientation, dissected and fixed o/n in 4% (w:v) PFA (171010, EMS) in 200 mM HEPES buffer (4-(2-hydroxyethyl)-1-piperazineethanesulfonic acid; Gibco, 15630-080) at pH 7.0 for 2 h at 4 °C. Fixed eyeballs were cryoprotected in a sucrose gradient (15% and 30%) and embedded in OCT (Tissue Tek, Sakura Finetek, 4583). Retinal sections (12 μm thick) were cut using a cryostat (Leica Microsystems).

### Ex-vivo retina explants and cell cultures

Neuroretinas were cultured in DMEM (Dulbecco’s Modified Eagle Medium; Gibco, 41966-029) with 1% glutamine (2 mM; 25030, Gibco), 1% penicillin-streptomycin (0.5 mg/ml; 11568876, Gibco) and 1 μM insulin (I2643, Sigma) for 18 h in a humidified incubator at 37 °C, 5% CO_2_ with the following agents: MNU (100, 500 and 1000 µg/ml; TR-M325815, Toronto Chemicals), DFP (3-Hydroxy-1,2-dimethyl-4(1H)-pyridone, 1 mM; 379409, Sigma-Aldrich) and olaparib (10 µM; S1060, Selleckchem). For flow cytometer experiments, *N* = 4 per treatment; for GFAP immunofluorescence, *N* = 4 per treatment; for ARR3 inmunofluorescence, *N* = 6-8 per treatment.

ARPE-19 (ATCC, CRL-2302) and ARPE-19 *mito-*QC cells were grown in DMEM: F12 (1:1) supplemented with 15% FBS, 1% glutamine (2 mM; 25.030, Gibco), and 1% penicillin-streptomycin (0.5 mg/ml; 11568876, Gibco) in a humidified incubator at 37 °C, 5% CO_2_. The selection of cells with the *mito-*QC reporter was achieved with Hygromycin B at 800 μg/ml (10453982, Gibco) [18]. For immunofluorescence detection, lysosomal pH measurement, and flow cytometer analysis, 30,000 cells per well were seeded in 24-well plates. For western blot analysis, 300,000 cells were seeded in 6 wells of culture plates. Cells were treated with different compounds: MNU (100, 500 and 1000 µg/ml; TR-M325815, Toronto Chemicals); DFP (3-Hydroxy-1,2-dimethyl-4(1H)-pyridone; 1 mM; 379409, Sigma-Aldrich); LLOMe (Leu-Leu methyl ester hydrobromide; 1 mM; L7393, Sigma); and olaparib (10 µM; S1060, Selleckchem). Cells were transfected overnight with 1 μg/ml tfGalectin-3 (Gal-3) plasmid (64149, Addgene) using Lipofectamine 3000 (L300015, Thermo Fisher). The medium was then changed and Geneticin/G418 (1 μg/ml; 11811-023, Gibco) selection was performed over 24 h. Next, GFP high-RFP cells sorted with a FACSaria Fusion Cell Sorter (Beckton-Dickinson) were expanded for the experiments.

To knock down expression of *PINK1, PRKN, BNIP3* and *BNIP3L*, ARPE-19 *mito*-QC were grown at 60–80% confluency in p100 plates, and then transfected with 60 pmol scramble, *PINK1, PRKN, BNIP3*, and *BNIP3L* coding siRNA (AM4611, s35166, s78529, s2061, s531927, Ambion-Life Technologies)). Following the manufacturer’s instructions (Invitrogen), siRNA and Lipofectamine™ RNAiMAX reagent (13788, Invitrogen) were diluted in Opti-MEM® medium (11058-021, Gibco). The diluted siRNA was transferred to the Lipofectamine™ RNAiMAX for liposome formation, and incubated for 10 minutes at room temperature. The complexes were added to the cells, which were then incubated in DMEM-F12 medium for 24 h in a humidified incubator at 37 °C, 5% CO_2_. Cells were then trypsinized, counted, and 40,000 cells plated in 24-well plates. After 4 h, cells were incubated with the corresponding compound for 18 h, after which mRNA levels were determined by qPCR and cell death levels measured by flow cytometry.

### Mitophagy assessment and immunofluorescence in cells and retina sampless

Mitophagy was assessed in ARPE-19 *mito*-QC cells. These cells were generated in the laboratory of Ian Ganley and ARPE-19 also come from Ian Ganley’s laboratory. Mycoplasma checks have been carried out every 6 months on the cell lines. DAPI staining (4’,6-diamino-2-phenylindole, 1 μg/ml; D9542, Sigma) was used to visualize the nuclei and mounting was with Prolong Diamond (P36961; Thermo Fisher). For immunofluorescence, cells were permeabilized with 0.1% (v:v) SDS or 0.3% (v:v) Triton X-100 (T9284, Sigma) and incubated with primary antibodies for 1 h at RT. The primary antibodies used were anti-γH2AX (1:100; ab22551, Abcam;) and anti-PAR (1:100; 4335-MC-100, Bio-Techne R&D Systems). After washing with PBS, tissues and cells were incubated in darkness for 1 h at RT with secondary antibodies (1/200; Alexa Fluor 647; Invitrogen, A11075) and DAPI. Cells were also incubated with LysoTracker Red DND-99 (1 μM; L7528, ThermoFisher;) for 15 min before fixation with 3.7% PFA. Cells were mounted with Prolong Diamond (P36961; Thermo Fisher).

Mitophagy was assessed in retinal sections and flat mounts with DAPI staining to visualize the nuclei and they were mounted with Vectashield (H-1000-10, Palex medical). For immunofluorescence, retinas were permeabilized with 0.3% (v:v) Triton X-100 (T9284, Sigma) and blocked with BGT (3 mg/ml BSA, 0.25% Triton X-100, 100 mM glycine in PBS) for 1 h. Retina samples were incubated with primary antibodies o/n at 4 °C. The following primary antibodies were used: anti-4-HNE (1:100; ab46545, Abcam); anti-GFAP (1:500; Z0334, DAKO); anti-TOMM20 (1:200: sc-11415, Santa Cruz Biotechnology); anti-cleaved-CASP3 (1:100; 9661, Cell Sig.), anti-ARR3/Cone Arrestin (1:200; AB15282, Millipore); anti-SAG/Visual arrestin (1:100; sc-166383, Santa Cruz); anti-PAR/pADPr (1:100; 4335-MC-100, Bio-Techne R&D Systems) and anti-AIF (1:100; 4642, Cell Signaling). After washing with PBS, retinal tissue was incubated for 1 h at RT in darkness with secondary antibodies (1/200; Alexa Fluor 488, Alexa Fluor 568, and Alexa Fluor 647; Invitrogen, A11011, A21247 and A11075) and DAPI. Retinal flat mounts were mounted with Fluoromount (100-01; Bionova) between 2 sealed coverslips, to allow the explant to be turned over depending on the layer of interest for imaging. Cryosections were mounted with Fluoromount and the coverslips sealed with nail polish.

TUNEL assay (DeadEnd™ Fluorometric TUNEL System; G3250; Promega) was used to detect apoptotic cells in cryosections following the manufacturer’s instructions. Briefly, once the primary antibody was washed, cryosections were incubated for 30 min with TUNEL buffer, after which the TUNEL reaction (1.9% TdT, 9.8% dNTPs, and 88.3% TUNEL buffer) was performed in darkness for 1 h at 37 °C. Saline-sodium citrate (SSC) provided in the kit (20X) was diluted to a concentration of 2X and added to cryosections to stop the TUNEL reaction.

### Fluorescence-based measurement of lysosomal pH

For acid lysosome staining, ARPE-19 cells were incubated with LysoTracker Red DND-99 (1 μM; L7528, ThermoFisher) for 15 min at 37 °C. To track fluctuation in lysosomal pH, LysoSensor Yellow/Blue DND-160 (50 μM; L7545, Thermo Fisher Scientific) was added to ARPE-19 cells incubated in DMEM without antibiotics for 90 min at 37 °C. Cells were then washed, fixed for 15 min with PFA 4%, and mounted with Prolong Diamond for fluorescence imaging and quantification.

### Flow cytometry and western blot

For flow cytometry analyses, cells were incubated with Trypsin-EDTA (0.05%; 25300054; Gibco) for 5 min at 37 °C. Each sample was centrifuged at 200 × g for 5 min and the resulting pellets were incubated with DAPI in all experiments to assess cell death. At least 5000 events were acquired in a Cytoflex S (Beckman Coulter). To determine mitochondrial superoxide production ARPE-19 cells were incubated with MitoSOX Red Mitochondrial Superoxide (2.5 μM; M36008, Invitrogen) for 15 min at 37 °C. Mean fluorescence intensity (MFI) parameter was used to measure the levels of superoxide. For the retina explants analyzed with MitoSOX, retinas were disaggregated after the culture with 300 µL Trypsin-EDTA (0.05%; 25300054; Gibco) in HBSS (14170-088; Gibco) during 10 min at 37 °C, trypsin-deactivated with 900 µL 10% FBS in HBSS and filtered with a cell strainer (352350; Falcon) before MitoSOX labelling.

Mitophagy was evaluated in ARPE-19 *mito*-QC cells. mCherry-PI (610/20), FITC-GFP (525/40), and PB450-DAPI (450/45) emission filters were used in the cytometer. For mitophagy assessment in *mito*-QC cells, a mCherry/GFP ratio of ~5% in control cells was set to define the high-mitophagy population. The percentage of high mitophagy cells was used to determine mitophagy levels. To measure viability, cells attached and non-attached were resuspended in 300 μL of media with DAPI (1 μg/ml; D9542, Sigma).

For western blot, we proceeded as previously described [19]. Briefly, ARPE-19 cells protein extracts were obtained in lysis RIPA buffer (R0278, Merck) supplemented with protease and phosphatase inhibitors. Protein concentration was measured with the Pierce BCA Protein Assay (23225; Thermo Scientific) following manufacturer’s instructions. Total protein extracts (12–45 µg) were supplemented with 5X loading buffer (4% glycerol, 0.5 M Tris-HCl pH 6.8, 8% SDS, 0.04% bromophenol blue, 5% β-mercaptoethanol) and resolved on Any kD Criterion TGX Precast Stain-free gels (5678124; Bio Rad). PVDF membranes were used for protein transference using a TransBlot Turbo Transfer System (Bio-Rad). Membranes were blocked with 5% milk in PBS-T (0.5% Tween-20 [1706531; Bio Rad] in PBS) for 1 h. Overnight incubations at 4 °C of the primary antibodies diluted 1:1000 in 5% BSA in PBS and 1 h incubations of the secondary antibodies diluted 1:2000–1:4000 in PBS-T were performed. Membranes were finally developed using Pierce ECL Western Blotting substrate (32106, Thermo Fisher) and x-ray film using a CURIX 60 Processor (AGFA).

### Image analysis and data quantification

All confocal images from a given experiment were acquired using the same laser intensity and photomultiplier settings to avoid variability or bias. Only nuclear DAPI staining was used to select the field to photograph. Images were acquired using a Leica TCS SP8 STED 3X and a Leica TCS SP5 multispectral confocal system. Images were processed with Fiji/Image J and Imaris software. BioRender has been used for experimental design schemes.

Unless stated otherwise, maximum projections of all z-stacks are displayed in representative images (z-step: 0.5 and 1 µm for cells and whole retina, respectively; 1 μm for cryosections). For retinal thickness measurements, each retinal layer was manually measured using the straight-line tool in ImageJ. Quantification of mean fluorescence intensity (MFI) was performed using unprocessed images at maximal projection. Cells positive for a given marker were quantified by manual counting plane by plane in a given z-stack. Biomarker-positive puncta and overall volume of positive immunostaining were determined for the markers 4-HNE, Mitophagy, LysoTracker Red, LysoSensor, and Gal-3, and were quantified using a manually designed Fiji-based plugin, which accounts for the 3D component. Briefly, a fluorescence intensity threshold is set to discriminate positive signal from background. Next, using a minimum voxel size threshold, the 3D objects counter tool [57] is used to detect the number of objects in a 3D confocal z-stack and to detect a volume of signal.

### Statistical analysis

All data are presented as mean values ± SEM. Statistical analyses were performed using GraphPad Prism software (GraphPad Software, Inc.). To assess differences between treatments, two-tailed Student’s *t*-tests were used. To evaluate differences between times and treatments two-way analyses of variance (ANOVA) were applied, and in cases of significant interactions differences between treatments for each timepoint were assessed using Fisher’s least significant difference (LSD) post-hoc tests. If criteria of normality and homoscedasticity were not met, non-parametric tests such as the Mann-Whitney *U*-test were used for two-group comparisons. For all tests the significance level was set at *p* < 0.05 (two-tailed). The number of animals used in each experiment is indicated in the corresponding figure legend.

The experiments with cells have been repeated at least 3 times. In animal experiments, a minimum of *N* = 4 has been used.

## Supplementary information

sup material

sup material wb

## Data Availability

The datasets generated during and/or analysed during the current study are available from the corresponding author on reasonable request.
