# Supplementary material for: Deferiprone protects against photoreceptor degeneration by inhibiting parthanatos
Source: Cell Death Dis. 2025 May 19;16(1):402. doi: 10.1038/s41419-025-07686-x (PMC12089389; doi:10.1038/s41419-025-07686-x)
Supplement: Supplementary file 1 — sup material [file 41419_2025_7686_MOESM1_ESM.pdf]

Contents of supplementary files:

"Sup Fig1": Supplementary figure 1: MNU induces caspase-3-independent cell death.

"Sup Fig2": Supplementary figure 2: Mitophagy is decreased in the ONL and increased on the OLM in mouse retinas treated with the lysosomal inhibitor leupeptin.

"Sup Fig3": Supplementary figure 3: Olaparib rescues MNU-induced cell death.

"Figures and legends": Supplementary figure legends

"Original WB": Full length uncropped original western blots used in Figure 5e.

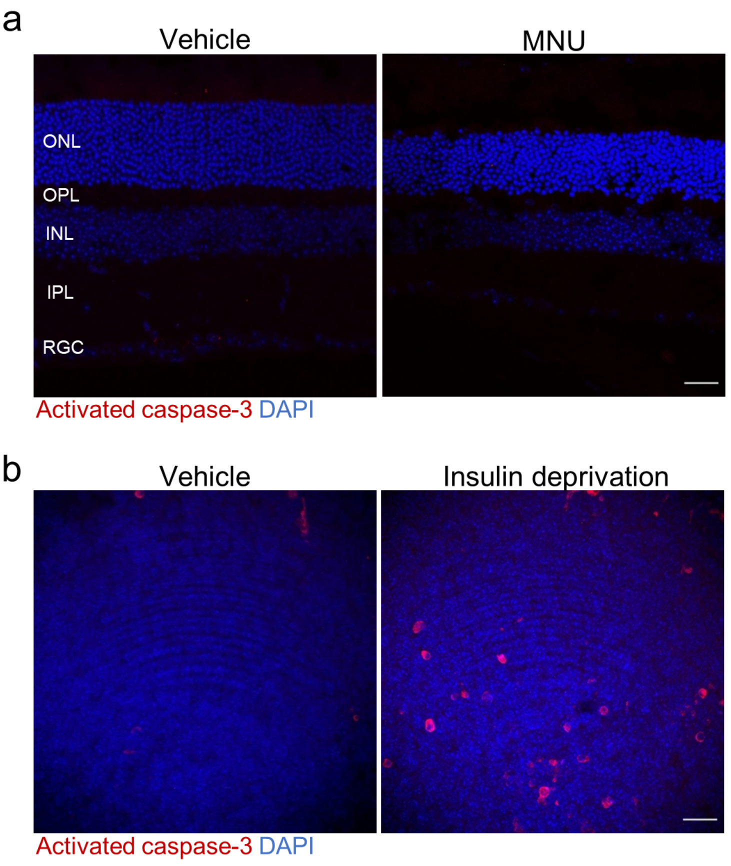
a
Vehicle
MNU
ONL
OPL
INL
IPL
RGC
Activated caspase-3 DAPI
b
Vehicle
Insulin deprivation
Activated caspase-3 DAPI

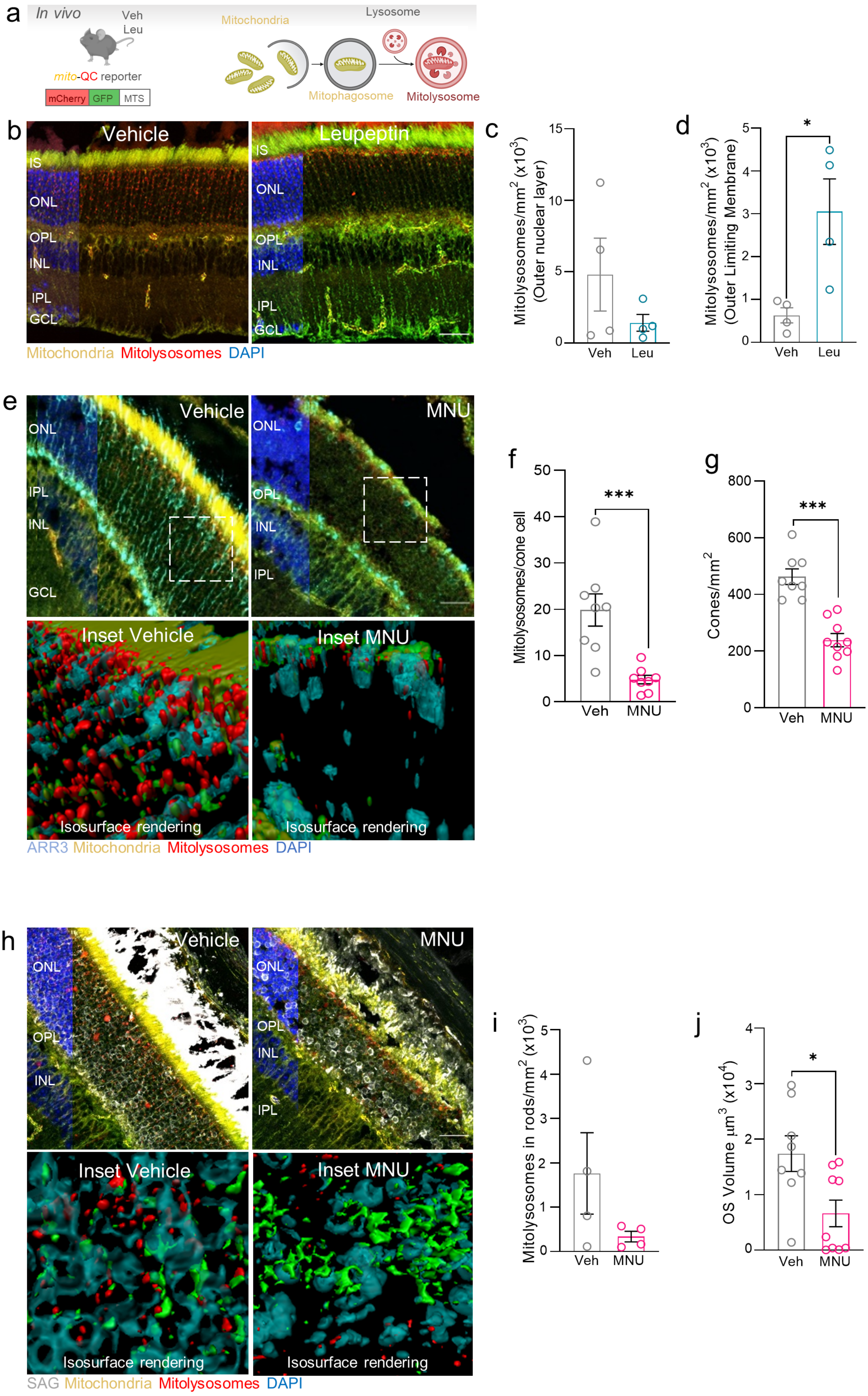

a
In vivo
Veh
Leu
mito-QC reporter
mCherry
GFP
MTS
Mitochondria
Lysosome
Mitophagosome
Mitolysosome
b
Vehicle
Leupeptin
IS
ONL
OPL
INL
IPL
GCL
Mitochondria Mitolysosomes DAPI
c
Mitolysosomes/mm^2 (x10^3) (Outer nuclear layer)
Veh
Leu
d
Mitolysosomes/mm^2 (x10^3) (Outer Limiting Membrane)
Veh
Leu
e
Vehicle
MNU
Inset Vehicle
Inset MNU
Isosurface rendering
ARR3 Mitochondria Mitolysosomes DAPI
f
Mitolysosomes/cone cell
Veh
MNU
g
Cones/mm^2
Veh
MNU
h
Vehicle
MNU
Inset Vehicle
Inset MNU
Isosurface rendering
SAG Mitochondria Mitolysosomes DAPI
i
Mitolysosomes in rods/mm^2 (x10^3)
Veh
MNU
j
OS Volume µm^3 (x10^4)
Veh
MNU

a

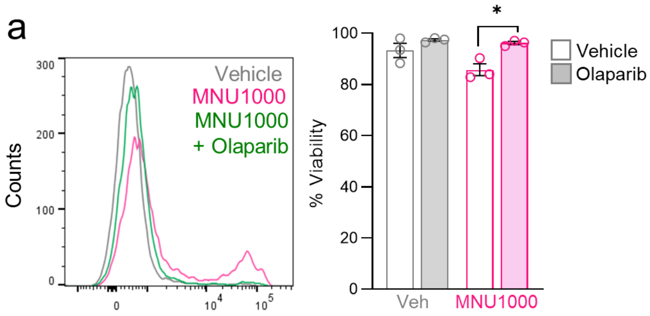

Counts
300
200
100
0
0
10^4
10^5
Vehicle
MNU1000
MNU1000
+ Olaparib
% Viability
100
80
60
40
20
0
*
Veh
MNU1000
Vehicle
Olaparib

Supplemental Figures and Legends

Deferiprone protects against photoreceptor degeneration by inhibiting parthanatos

Beatriz Villarejo-Zori[1#*], Juan Zapata-Muñoz[1*], Elena Sierra-Filardi[1], Ignacio Ramírez-Pardo[1,2], Lambert Montava-Garriga[3], Ian G Ganley[3], and Patricia Boya[1,4#]

[1]*Department of Cellular and Molecular Biology, Centro de Investigaciones Biológicas Margarita Salas, CSIC, Madrid, Spain.*

[2]*Department of Medicine and Life Sciences, Universitat Pompeu Fabra (UPF), CIBERNED, Barcelona 08003, Spain*

[3]*MRC Protein Phosphorylation and Ubiquitylation Unit, University of Dundee, Dundee DD1 5EH, UK.*

[4]*Department of Neuroscience and Movement Science, University of Fribourg, Chemin. du Musée 14, 1700 Fribourg, Switzerland.*

*Shared first autorship

[#]Correspondence to patricia.boya@unifr.ch or bvillarejo@cib.csic.es

Supplemental Figures

Sup. Figure 1. MNU induces caspase-3-independent cell death. Immunostaining of activated caspase-3 (red) and DAPI (blue) in (a) retinal cryosections from mice treated with MNU (60 mg/kg) or vehicle for 24 h and (b) in flat mounts from embryonic day 13.5 retinas subjected to insulin deprivation, as a positive control for immunostaining of activated caspase-3. Scale bars: 25 µm.

Sup. Figure 2. Mitophagy is decreased in the ONL and increased on the OLM in mouse retinas treated with the lysosomal inhibitor leupeptin. (a) Experimental design. (b) Representative *mito*-QC retina cryosections of mice treated with Leupeptin (40 mg/kg) or vehicle (n= 4 per group) for 18 h. (c) Quantification of red dots (mitophagy) in ONL and (d) OLM. (e) Representative images of mitolysosomes inside cone cells of *mito*-QC retina sections labeled with anti-ARR3 (cyan) antibody. White arrowheads indicate red dots inside cones in the isosurface rendering with Imaris. (f) Quantification of red dots within cones and (g) and the number of cones per mm^2 (g). (h) Representative images of mitolysosomes inside rods of *mito*-QC retina sections labeled with anti-SAG (gray) antibody. (i) Quantification of red dots within rods and (j) volume of outer segments (μm^3). Circles in graphs represent individual mice. All data are expressed as the mean ± SEM. *p <0.05; **p <0.01; ***p <0.001; ***p <0.001. . Statistical analyses were performed using a two-tailed Student´s *t*-test. Scale bars: 25 µm.

Sup. Figure 3. Olaparib rescues MNU-induced cell death. (a) Flow cytometry quantification of viability based on DAPI intensity in ARPE-19 *mito*-QC cells treated with MNU (1000 µg/ml) and/or olaparib (10 µM) for 18 h **(left)**, and representative histogram of the cell populations **(right)**. Dots represent different cell experiment. Data are expressed as the mean ± SEM. *p <0.05. Statistical analyses were performed using a two-tailed Student´s *t*-test.
