# Supplementary figures and images for: Deferiprone protects against photoreceptor degeneration by inhibiting parthanatos
Source: Cell Death Dis. 2025 May 19;16(1):402. doi: 10.1038/s41419-025-07686-x (PMC12089389; doi:10.1038/s41419-025-07686-x)

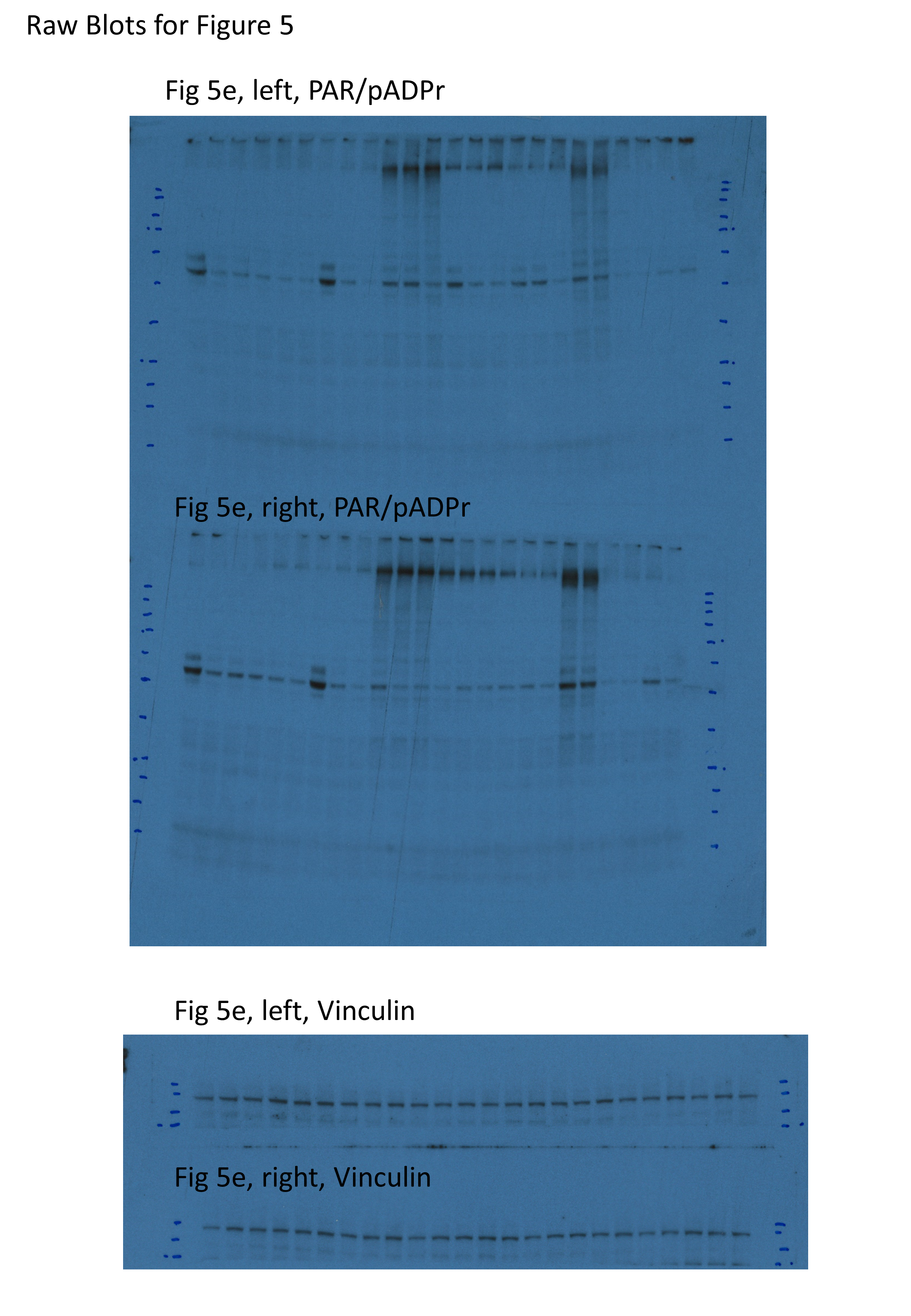

Supplement: Supplementary file 2 — sup material wb [file 41419_2025_7686_MOESM2_ESM.tif]
